# Acoustofluidic Tweezers Integrated with Droplet Sensing Enable Multifunctional Closed‐Loop Droplet Manipulation

**DOI:** 10.1002/advs.202409394

**Published:** 2024-11-11

**Authors:** Mingyang Sui, Huijuan Dong, Guanyu Mu, Zhen Yang, Ye Ai, Jie Zhao

**Affiliations:** ^1^ State Key Laboratory of Robotics and System Harbin Institute of Technology Harbin 150001 China; ^2^ Institute of Orthopedics Chinese PLA General Hospital Beijing Key Laboratory of Regenerative Medicine in Orthopedics Key Laboratory of Musculoskeletal Trauma & War Injuries PLA Beijing 100853 China; ^3^ Pillar of Engineering Product Development Singapore University of Technology and Design Singapore 487372 Singapore

**Keywords:** acoustic detection, closed‐loop system, droplet manipulation, slanted finger interdigital transducers, surface acoustic waves

## Abstract

Droplet manipulation technologies with surface acoustic waves attract significant attention for applications in fluid handling and bioanalysis. However, existing technologies face challenges in automation, precision, and functional integration, limiting broader applications. In this work, a highly integrated droplet‐sensing acoustofluidic tweezer is developed, incorporating orthogonally arranged slanted finger interdigital transducers and a custom‐designed control and detection circuit system. Using a single acoustic device, this tweezer enables switchable acoustic droplet manipulation and detection, providing multifunctional closed‐loop manipulation of on‐chip microliter‐scale droplets. The platform takes advantage of the wideband frequency response characteristics of the transducers, along with an automated droplet detection algorithm, enabling high‐precision detection of central positions, edge positions, contact diameters, and the number of droplets. With this feedback, automated closed‐loop control of various droplet manipulation functions, including transportation, merging, mixing, splitting, and internal particle enrichment, is achieved for the first time on a single acoustic platform. This significantly enhances the precision, efficiency, and fault tolerance of the manipulation process. This droplet‐sensing acoustofluidic tweezer provides an innovative acoustic solution for droplet manipulation technologies in fields such as fluid processing and biosensing, demonstrating significant application potential.

## Graphical abstract

A highly integrated droplet‐sensing acoustofluidic tweezer enables switchable surface acoustic wave‐based droplet manipulation and detection. This advanced acoustic platform provides closed‐loop control of multiple droplet functions, including droplet transportation, merging, mixing, splitting, and particle enrichment within droplets, significantly enhancing precision, efficiency, and fault tolerance in fluid processing and biosensing applications.

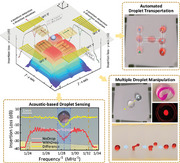

## 1 Introduction

Controllable droplet manipulation is crucial in many applications, including chemical reactions,^[^ ^1^, ^2^ ^]^ bioanalysis,^[^ ^3^, ^4^ ^]^ real‐time detection,^[^ ^5^, ^6^ ^]^ and drug delivery.^[^ ^7^, ^8^ ^]^ Among these, lab‐on‐a‐chip technologies based on various fluid handling principles such as body force,^[^ ^9^, ^10^ ^]^ electricity,^[^ ^11^, ^12^, ^13^, ^14^, ^15^ ^]^ magnetism,^[^ ^16^, ^17^ ^]^ light,^[^ ^18^, ^19^ ^]^ and acoustics^[^ ^20^, ^21^, ^22^, ^23^, ^24^, ^25^ ^]^ have been widely applied. Particularly, acoustic technology has emerged as a promising avenue for droplet manipulation, known as acoustofluidic tweezers,^[^ ^26^ ^]^ due to its low loss,^[^ ^27^ ^]^ reusability,^[^ ^28^ ^]^ high biocompatibility,^[^ ^29^, ^30^ ^]^ and independence from the electrical or optical properties of the target,^[^ ^31^, ^32^ ^]^ without the need for complex surface structures or droplet pre‐treatment.^[^ ^33^, ^34^ ^]^

However, most existing acoustofluidic tweezer technologies for droplet manipulation are designed to perform specific functions,^[^ ^35^, ^36^, ^37^, ^38^ ^]^ and acoustofluidic platforms capable of integrating multiple functions are extremely rare. Additionally, both single‐function and multifunction acoustic systems generally rely on open‐loop control, which leads to deficiencies in precision and automation.^[^ ^39^, ^40^ ^]^ For acoustofluidic tweezer technologies, minor scratches or impurities on the chip surface, uneven surface treatments,^[^ ^41^ ^]^ changes in external temperature,^[^ ^42^ ^]^ and slight variations in the physical properties of droplets^[^ ^43^ ^]^ can all lead to inconsistent and unpredictable performance in droplet manipulation. Although using arrays of transducers provides some controllability over the droplet path,^[^ ^44^ ^]^ the inherent limitations of open‐loop systems, such as low fault tolerance and limited automation, still pose significant technical challenges.^[^ ^45^ ^]^

Additionally, existing droplet microfluidic tweezers primarily rely on electrical impedance mechanisms to provide feedback on droplet position, which are commonly used in electro‐wetting‐based digital microfluidic systems.^[^ ^46^, ^47^ ^]^ However, this method is less compatible with acoustofluidic tweezers because it requires emphasizing the electrical properties of the droplets and setting up additional on‐chip electrodes.^[^ ^48^, ^49^ ^]^ Although capacitive feedback methods for droplet position exist,^[^ ^50^ ^]^ their application is limited to designs with single, fixed functions; otherwise, the additional electrodes interfere with the droplet manipulation capabilities of the acoustofluidic tweezers. While closed‐loop control methods that utilize image‐based recognition can enhance control precision and automation by providing feedback,^[^ ^51^, ^52^ ^]^ these methods require specific lighting conditions and additional equipment components, which are not conducive to system integration. Even acoustic‐based feedback methods face challenges such as the need for extra acoustic devices and the inability to integrate multiple functions.^[^ ^53^, ^54^ ^]^ Overall, existing acoustic technologies have yet to fully meet the requirements for automation, precision, and multifunctional integration, which restricts their widespread application and practicality in droplet manipulation.

To address these challenges, we have developed a highly integrated *droplet‐sensing acoustofluidic tweezer* (DSAT), which includes orthogonally opposed slanted finger interdigital transducers (SFITs) and a specially designed control and detection circuit system. The DSAT simultaneously integrates surface acoustic wave (SAW)‐based droplet actuation and sensing capabilities on a single acoustic device, with programmatic control allowing seamless switching between these two modes for multifunctional closed‐loop droplet manipulation. To our knowledge, it is the first platform to achieve this functionality based on acoustic principles. Taking advantage of the broad frequency response characteristics of the SFITs and a custom‐developed automated droplet detection algorithm, the system can precisely detect the center positions, edge positions, contact diameters, and the number of droplets. Using this feedback, droplets can be automatically manipulated to perform microfluidic tasks without manual observation, pre‐calibration, preset surface structures, or visual setups. Furthermore, DSAT is the first platform to integrate acoustic methods to achieve five key droplet manipulation functions, including transport, merging, splitting, mixing, and internal particle enrichment, thereby fully meeting the needs of the fluid processing^[^ ^55^ ^]^ and biosensing^[^ ^56^ ^]^ fields.

Specifically, in this paper, the system composition and working principles of DSAT were detailed, including the SFITs device, the droplet actuation module, and the droplet detection module. The droplet sensing performance of DSAT, including the accuracy and repeatability of position detection, has been experimentally validated. By integrating the actuation and detection modes, automated closed‐loop control of droplet transport on the chip was achieved. Furthermore, experiments have demonstrated that real‐time feedback on droplet position and quantity significantly enhances the precision and fault tolerance of various droplet manipulation functions. This droplet‐sensing acoustofluidic tweezer opens new avenues for multifunctional automated closed‐loop droplet manipulation technologies in practical applications, presenting significant viable prospects.

## 2 Working Principles of the Droplet‐Sensing Acoustofluidic Tweezers (DSAT)

**Figure 1:**
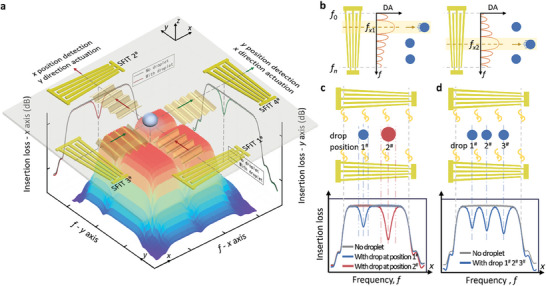
Schematic and working principles of the droplet‐sensing acoutofluidic tweezers (DSAT) for multifunctional closed‐loop droplet manipulation. a) Schematic of the SFITs device of the DSAT. b) Schematic of the selective droplet actuation using SFITs. The curves represent the amplitude characteristics of the waves generated by the SFITs at different excitation frequencies, where “DA” on the *y*‐axis refers to “Displacement Amplitude”, indicating the amplitude of SAWs. c) Schematic of the detection principle for the center and edge positions of a single droplet. Here, Insertion Loss refers to the energy loss in SAW devices as the wave travels from the transmitter to the receiver, quantified by the ratio of output power to input power, typically expressed in decibels. d) Schematic of the detection principle for the number and positions of multiple droplets. The characteristic curves in c and d were obtained by simulations using the Coupling‐of‐Modes (COM) model^[^ ^65^ ^]^ (Note S1, Supporting Information).

Travelling surface acoustic waves (TSAWs) can be generated by exciting the interdigital transducers on a piezoelectric substrate. These waves propagate along the substrate surface and couple into the droplets in their path, actuating internal acoustic streaming,^[^ ^57^ ^]^ movement,^[^ ^58^ ^]^ or ejection,^[^ ^59^, ^60^ ^]^ depending on the amplitudes of the TSAWs. This is the fundamental principle by which the acoustofluidic tweezers manipulate droplets. Here, the key acoustic component of the proposed DSAT consists of two pairs of orthogonally aligned SFITs, as shown in **Figure** **1**a. The finger periods of the SFITs vary at different positions along the aperture, giving them broadband frequency responses. Leveraging this characteristic, selective actuation of the on‐chip droplets and the sensing of their presence, number, and positions can be achieved using the DSAT.

For droplet actuation, as shown in Figure 1b, high‐amplitude, narrow‐band TSAW beams can be generated at specific positions using the SFIT excited at specific frequencies, actuating the droplets along their path. In contrast, TSAWs generated at other positions have lower amplitudes, which are insufficient to actuate the droplets^[^ ^29^, ^61^, ^62^, ^63^ ^]^ (numerical simulation results detailed in Note S2, Supporting Information). By adjusting the excitation frequency, it is possible to precisely change the position of the generated high‐amplitude TSAW beams, thus enabling selective actuation of the droplets. For droplet sensing, as illustrated in Figure 1c, a pair of opposing SFITs forms a broadband SAW resonator,^[^ ^64^ ^]^ with its frequency response shown as a gray curve. When droplets are located on the wave propagation path of the resonator, acoustic wave loss induces “insertion loss decrease zone” on the frequency response curve, depicted as a blue curve. Different positions and volumes of the droplets alter the “insertion loss decrease zones” on the frequency response curve. Specifically, differences in position cause shifts in frequency, while differences in volume (which affect the contact diameter and edge positions) change the width of these “insertion loss decrease zones”, as shown by the red curve in Figure 1c. Furthermore, the presence of multiple droplets results in multiple “insertion loss decrease zones” on the frequency response curve (Figure 1d). In summary, comparing the frequency response of the SFITs resonator with and without droplets allows for the accurate detection of the presence of droplets on the substrate, their number, edge positions, contact diameters, and central positions.

Integrating the principles of droplet actuation and sensing described above, the DSAT utilizes two pairs of SFITs to generate narrow TSAW beams in the *x*± and *y*± directions. This enables the DSAT to manipulate droplets on a 2D plane, performing functions such as droplet transport, merging, splitting, mixing, and internal particle enrichment. Additionally, by analyzing the frequency responses of SFIT 1^#^2^#^ and SFIT 3^#^4^#^, the number of droplets and their positions along the *x* and *y* axes can be detected. This results in an acoustofluidic tweezers system capable of sensing droplets, integrating both droplet actuation and detection modes, and allowing for mode switching based on operational requirements. These capabilities provide the acoustofluidic tweezers with intelligent and automated characteristics, enabling closed‐loop droplet manipulation.

## 3 Integrated DSAT System for Closed‐Loop Droplet Manipulation

**Figure 2:**
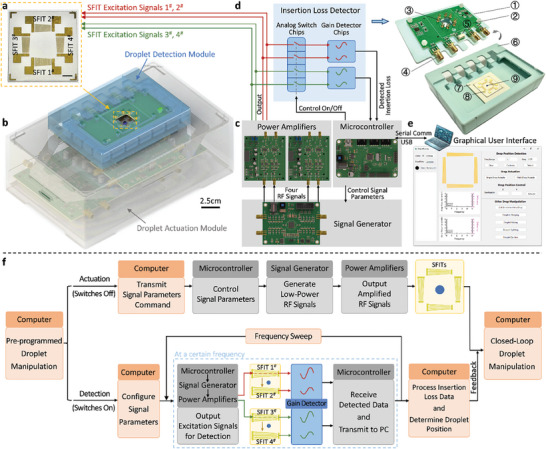
System configuration of the droplet‐sensing acoutofluidic tweezers (DSAT). a) Photo of the fabricated 2D SFITs device. b) Rendered image of the DSAT system, including the SFITs device, droplet actuation module (gray casing, 20 cm × 13 cm × 7 cm), and droplet detection module (blue casing, 12 cm × 9 cm × 2 cm). c) Circuit configuration and signal transmission relationships of the droplet actuation module, including the microcontroller, signal generator, and power amplifiers. d) Schematic of the circuit of the droplet detection module displayed on the left, and the photo of the instrument integrated with the droplet detection module and the SFITs device shown on the right. The components include: 1) top cover of the casing, 2) insertion loss detector circuit board, 3) spring contacts, 4) RF interfaces, 5) detection signal interface, 6) base of the casing, 7) base for placing the SAW device, 8) SFITs device, 9) droplet to be manipulated. e) GUI of the control system, used for human‐machine interaction, running the host computer's control program, and initiating/stopping droplet manipulation commands. f) Schematic illustrates the control and execution flowchart of the closed‐loop droplet manipulation system.

Here, we developed an integrated DSAT system that incorporates a 2D SFITs device (**Figure** **2**a) and integrates both a droplet actuation module and a droplet detection module. This system has overall dimensions of 20 cm × 13 cm × 9 cm (Figure 2b), enabling the use of DSAT technology for multifunctional closed‐loop droplet manipulation in a portable device. The SFITs device consists of a piezoelectric substrate with orthogonally aligned SFITs deposited on it, labeled SFIT 1^#^2^#^3^#^4^#^. The resonant frequency bandwidths for pairs of SFITs, determined through testing, are 24.2 to 32.3 MHz for SFIT 1^#^2^#^ and 22.6 to 30.7 MHz for SFIT 3^#^4^#^. Each SFIT was designed with an aperture of 6 mm and an effective aperture of ≈1 mm (Note S3, Supporting Information). This configuration allows for the generation of narrow TSAW beams ≈1 mm wide at positions corresponding to the excitation frequencies within the 6 mm × 6 mm area formed by the two pairs of SFITs,^[^ ^63^, ^66^, ^67^ ^]^ enabling both droplet actuation and sensing.

As shown in Figure 2c, the droplet actuation module connects to a host computer via a microcontroller, which controls a signal generator to produce and amplify radio frequency (RF) signals. It can independently output four channels of adjustable RF power signals with frequencies from 1 to 70 MHz and amplitudes from 0 to 20 V, which are applied to SFIT 1^#^2^#^3^#^4^#^. This setup allows for the excitation of TSAWs with varied amplitudes at different positions on the SFITs by controlling the on/off state of different channels and adjusting their frequencies and amplitudes according to the requirements of droplet manipulation. Meanwhile, as shown in Figure 2d, the droplet detection module receives signals from the droplet actuation module via four RF interfaces and transmits them to the SFITs. This module uses gain detector chips to detect the frequency response characteristics of the SFIT resonators, enabling droplet sensing. The microcontroller controls the analog switches to toggle between actuation and detection modes. In actuation mode, the RF signals are directly applied to the SFITs, generating TSAWs to actuate the droplets. In detection mode, the RF signals are transmitted to the SFITs to generate waves for droplet detection while being analyzed by the gain detector chips to obtain the frequency response of the SFIT resonators (detailed in Note S4, Supporting Information).

The DSAT system achieves closed‐loop manipulation of droplets through coordinated control between the microcontroller and the host computer. The microcontroller receives commands from the host computer to generate RF signals with varying frequencies and amplitudes for droplet actuation or sensing. The host computer provides human‐machine interaction functionality through a graphical user interface (GUI) designed using Qt software (Figure 2e). Users can set parameters such as serial ports, baud rate, frequency sweep range, step size, and target position coordinates, and initiate or stop droplet manipulation. The built‐in control logic of the system automatically manages signal distribution, data collection, and droplet position analysis, ensuring that droplet operations are carried out according to preset instructions. This coordinated control strategy supports automated droplet detection and allows for flexible switching between the droplet actuation and detection modes, achieving multifunctional droplet manipulation.

Figure 2f illustrates the closed‐loop droplet manipulation process based on the DSAT system configuration. The operator initiates the droplet manipulation program via the GUI, and the host computer automatically carries out actuation or detection commands according to a preset sequence. In actuation mode, the droplet actuation module generates the required high‐amplitude RF signals (13–20 V), which are applied to the SFITs to actuate the droplets.

In detection mode, the gain detector chips are connected to the system through the closed switches. The host computer controls the droplet actuation module to output low‐amplitude RF reference signals (3 V) within the set frequency range and step size, applied to SFIT 1^#^. These signals propagate through the acoustic resonator formed by SFIT 1^#^2^#^, and are collected by the gain detector chip on SFIT 2^#^ as detection signals, reflecting the insertion loss between SFIT 1^#^2^#^. The same process is applied to SFIT 3^#^4^#^. After the frequency sweep, the host computer collects and analyzes the insertion loss data to obtain the frequency responses of the SFITs. By comparing these responses with those obtained without droplets and processing them through algorithms, the positions of the droplets in the *x* and *y* directions are determined. Based on the feedback from droplet detection, the closed‐loop droplet manipulation process can be automatically carried out.

## 4 Results

### 4.1 Acoustofluidic‐Based Droplet Sensing

**Figure 3:**
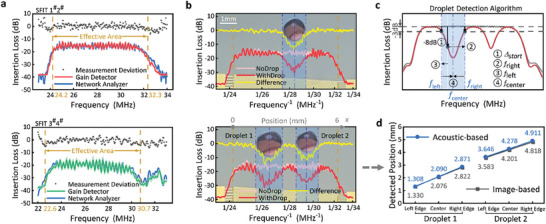
Frequency response detection using DSAT and droplet sensing algorithm. a) Detected frequency response curve of the SFITs device. Each test was repeated ten times, collecting data at 221 frequency points per test. b) Frequency response without and with the droplet. Here, the light red and red curves represent the frequency responses of the SFITs without and with droplets, respectively, and the yellow curve represents the differences in frequency response. c) Schematic of the center‐expansion algorithm used to determine the number and positions of the droplets. d) Comparison of acoustic‐based and image‐based methods for detecting the positions of Droplet 1 and Droplet 2 shown in b.

The sensing capability of the DSAT significantly impacts the precision and efficiency of automated closed‐loop droplet manipulation. Therefore, we first evaluated the performance of the DSAT in detecting the number and positions of droplets. Acoustofluidic‐based droplet detection is achieved by analyzing the frequency responses of the SFITs. We carried out frequency response tests on SFIT 1^#^2^#^ within the 23–34 MHz range and on SFIT 3^#^4^#^ within the 22–33 MHz range, with a step size of 0.05 MHz, using the DSAT. The detection results were highly consistent with the reference values obtained using a network analyzer (**Figure** **3**a). The average deviation for frequency response detection of SFIT 1^#^2^#^ was 1.63 dB, and 1.85 dB for SFIT 3^#^4^#^ (representing precision), with standard deviations of 0.51 and 1.03 dB (representing repeatability), respectively. Within the resonant frequency bandwidth, the average deviations were even smaller, reaching 1.20 and 1.37 dB, respectively. These results confirm the high precision and repeatability of DSAT in frequency response detection, ensuring its accuracy and reliability in droplet detection.

Droplet positions can be determined by analyzing the frequency responses of the SFITs (Figure 3b). During detection, the frequency response of the SFITs is first measured without any droplets present, in a process known as calibration. After placing the droplets, the frequency response is measured again to produce a differential frequency response curve. Since the finger periods change uniformly relative to the position on the SFITs, the frequency points on the curve, denoted as *f*, can be converted into positional coordinates through a function relating frequency to position, written as:^[^ ^62^ ^]^

(1) $$\;P_{i}=\frac{A}{\frac{1}{f_{L,i}}-\frac{1}{f_{H,i}}}\;\left(\frac{1}{f_{i}}-\frac{1}{f_{H,i}}\right)\;\mathrm{for}\;i\in \left\{x,y\right\}\;$$

where *P_i_* represents the calculated position along the axis *i* (*x* or *y*), *f_i_* represents the current frequency, and *A* represents the aperture. *f_L_*_,_ *_i_* and *f_H_*_,_ *_i_* respectively represent the lower and upper frequency limits for the SFITs on axis *i*. The positional coordinates are inversely proportional to the frequency; therefore, reciprocal coordinates are used for the abscissa of the frequency response curve to correspond to the actual droplet positions. The centers and edges of the “insertion loss decrease zones” on the curve correspond to the center and edge positions of the droplets, respectively. In this paper, we introduce a center‐expansion algorithm to automate droplet detection within the system. This algorithm automatically analyzes and identifies the “insertion loss decrease zones” in the frequency response differences, accurately determining the number and positions of droplets (Figure 3c). The algorithm starts at the low‐frequency end of the band, locating the first frequency point where the difference exceeds −8 dB, and extends toward both lower and higher frequencies until reaching points where the difference is −3 dB, marking the droplet edges. The droplet center is determined by the midpoint between these edge positions. The search continues beyond this droplet on the higher frequency side to locate the next point where the difference exceeds −8 dB, determining the number and positions of all droplets (see Note S5, Supporting Information). Using this algorithm to analyze the frequency response difference data shown in Figure 3b, the acoustofluidic‐based detection results for the edges and center positions of the Droplet 1 and Droplet 2 were obtained (Figure 3d). These results were very close to the image‐based identification results, validating the effectiveness of the DSAT system and algorithm in automatically detecting droplet positions.

**Figure 4:**
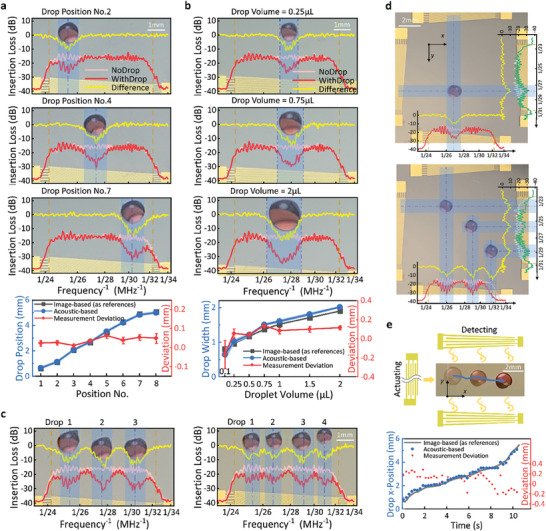
Droplet sensing characteristics of the DSAT. a) Results for the detection of droplet center positions and comparison with image‐based detection results. The detected frequency response curves for positions No.2, 4, and 7 are shown (additional details available in Note S6, Supporting Information). b) Results for the detection of edge positions and contact diameters of the droplets with different volumes (0.1, 0.25, 0.5, 0.75, 1, 1.5, 2 µL) and comparison with image‐based detection results. The detected frequency response curves for droplet volumes of 0.25, 0.75, and 2 µL were shown (additional details available in Note S7, Supporting Information). c) Detection results of the frequency response of multiple droplets. Detection of three 0.5 µL droplets and four 0.3 µL droplets, respectively. In the frequency response graphs of a, b, and c, blue dashed and double‐dashed lines indicate detected droplet centers and edge positions, respectively, with the blue areas representing the contact diameters. d) Detection results of the 2D positions of the droplets. e) Experimental setup and results for detecting the positions of moving droplets. To detect the position of continuously moving droplets, an SAW device different from the previously mentioned 2D SFITs was employed. This device is equipped with a uniform spaced interdigital transducer to continuously drive the droplet along the *x*‐direction, while a pair of opposing SFITs was used to detect the *x*‐position of the droplet every 260 ms (this device was used only in this experiment). The data graph shows the comparison between droplet positions identified by frame‐by‐frame image‐based analysis and those detected acoustically.

Next, SFIT 1^#^2^#^ of the DSAT was used to automatically detect the *x*‐direction center positions of droplets to evaluate its detection performance (**Figure** **4**a; Note S6, Supporting Information). The results were highly consistent with those obtained from the image‐based method, with an average deviation of 0.038 mm and a standard deviation of 0.015 mm. Subsequently, the edge positions and contact diameters of the droplets were measured (Figure 4b). The results showed that for droplets larger than 0.25 µL, the average deviation for contact diameter detection was 0.090 mm with a standard deviation of 0.031 mm. For edge position detection, the average deviation was 0.052 mm with a standard deviation of 0.024 mm. For droplets smaller than 0.25 µL, the accuracy and repeatability of contact diameter detection significantly decreased, posing a risk of failure in automatic recognition (see Note S7, Supporting Information). Consequently, we define the sensitivity threshold for droplet size detection of our developed DSATs as a minimum detectable volume of 0.25 µL, with a contact diameter of ≈0.96 mm. Moreover, the maximum detectable droplet volume is limited by the aperture area of the SFITs, rather than the detection system itself. Furthermore, the number and positions of multiple droplets were successfully detected using DSAT (Figure 4c). Due to the limitations of the aperture area, the platform can detect a maximum of four 0.3 µL droplets. It is noted that when two droplets are positioned too closely, the narrow TSAW beam propagating between them is attenuated by both droplets, which may cause the detection algorithm based on a ‐3 dB threshold to fail, mistakenly identifying two droplets as one. Experiments have demonstrated that maintaining a minimum operational distance of 0.2 mm between droplets can effectively prevent such identification errors.

Additionally, SFIT 3^#^4^#^ was used to detect the center positions of droplets in the *y*‐direction, with an average deviation of 0.066 mm and a standard deviation of 0.020 mm (Note S8, Supporting Information). The precision and repeatability in the *y*‐direction were slightly lower than those in the *x*‐direction, primarily due to the varying propagation performance of the TSAWs on the substrate in the *x* and *y* directions. Despite these differences, the accuracy and reliability of the detection still meet the requirements for droplet manipulation and effectively support the 2D positioning of both single and multiple droplets on the substrate (Figure 4d). Overall, these experimental results demonstrate that the DSAT exhibits high precision and good repeatability in measuring the center positions, edge positions, and contact diameters of droplets. Furthermore, the resolution and measurement time of the acoustic detection depend on the frequency sweep range and step size. Smaller steps result in longer measurement times and higher resolution. When measured with a step size of 0.05 MHz, the resolution varies between 0.0273 and 0.0503 mm depending on the finger periods of the SFITs (for details, see Note S9, Supporting Information). The measurement time for 1D detections is ≈260 ms, and for 2D detections, it is ≈450 ms. These short measurement times meet the real‐time and rapid response requirements of closed‐loop droplet manipulation.

In addition to the detection of stationary droplets discussed above, experiment to detect the positions of moving droplets was also carried out. The droplet continuously moved in the *x*‐direction actuated by the TSAWs, with the *x*‐position being detected every 260 ms. The detection results showed good consistency with reference values obtained through the image‐based recognition (Figure 4e), with an average deviation of 0.146 mm and a maximum deviation of 0.287 mm. Compared to the detection of stationary droplets, the accuracy was reduced due to the droplets being in motion during the frequency sweep detection, and the waves used for actuation possibly interfering with the detection. Therefore, to ensure higher accuracy, subsequent closed‐loop controls will implement an alternating control strategy between actuation and detection, ensuring that detection occurs when the droplets are stationary.

### 4.2 Closed‐Loop Droplet Transport Based on Droplet Position Detection

The integration of DSAT's droplet sensing capability with the actuation ability of the acoustofluidic tweezers enables closed‐loop manipulation of droplets. In this work, the DSAT applies RF power signals with amplitudes ranging from 13 to 20 V to the SFITs to actuate droplets. Different signal amplitudes correspond to different actuation modes: at 13 V, the SAWs couple into the droplets, inducing internal acoustic streaming, which is used for droplet mixing or particle enrichment; at 14–17 V, the SAWs drive the droplets to move, facilitating droplet transport or the merging of different droplets; at 20 V, the intensity of the SAWs is sufficient to actuate droplet ejection, thereby achieving droplet splitting.

**Figure 5:**
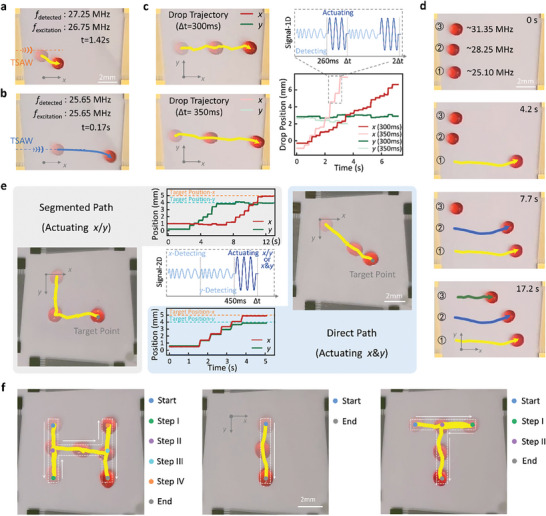
Closed‐loop droplet transport using DSAT. a) Droplet trajectory when the driving frequency is uncalibrated, offset by 0.5 MHz from the center frequency. Here the amplitude of the excitation signal, *V*_pp_, was set at 15 V. b) Droplet trajectory after calibrating the driving frequency using the DSAT, where the driving frequency was equal to the center frequency. *V*_pp_ = 15 V. c) Trajectories and corresponding time‐position relationships for droplet movement using frequency‐synchronized stepping driving method at Δt of 300 and 350 ms, respectively. *V*_pp_ = 15 V. Within the dashed box, the RF signal schematics for two control periods were illustrated, including the sweep‐frequency low‐amplitude signals used for droplet detection and the fixed‐frequency high‐amplitude signals used for droplet actuation. d) Sequential selective driving of multiple droplets. *V*_pp_ = 15 V. e) Two strategies for controlling droplet transport on the plane, including the segmented path strategy, where *V*_pp_ was set to 15 V, and the direct path strategy, where *V*_pp_ was dynamically adjusted between 14 and 15.5 V. Here, the droplet was first placed near the origin, then the DSAT automatically detected its position and drove it to the predetermined target position at coordinates (*x* = 4, *y* = 5). f) Automated droplet transport along “H”, “I”, “T” trajectories under position feedback closed‐loop control.

First, the process of driving droplets with the acoustofluidic tweezers based on SFITs will be optimized using DSAT's droplet sensing capability. Droplets can be driven by narrow TSAW beams generated by SFITs, which require precise alignment between the center of the droplet with the TSAW beam. Therefore, the excitation frequency must be adjusted to the center frequency corresponding to the center position of the droplet, ensuring the droplet moves effectively forward along the wave propagation direction. In practice, even a slight deviation of just 0.5 MHz from the center frequency in the excitation frequency can result in slow movement and lateral drift of the droplet, potentially causing it to stop moving (**Figure** **5**a). Traditional droplet manipulation techniques using acoustofluidic tweezers generally involve manual observation or preset markers for frequency adjustment,^[^ ^40^, ^61^, ^62^, ^68^, ^69^ ^]^ which are complex, inefficient, and challenging to achieve precise adjustment. However, the DSAT proposed in this work can automatically detect the center position of the droplet and determine the center frequency, ensuring precise frequency calibration. When the excitation frequency is accurately set to the center frequency, the droplet can move quickly and straight, traveling long distances with minimal lateral drift (Figure 5b).

However, continuously driving droplets at the center frequency may lead to issues such as lateral drift or changes in movement velocity due to uneven substrate surface treatments or variations in droplet shape, complicating the driving process. To address these problems, here we introduced a novel “frequency‐synchronized stepping drive method” using DSAT, where the driving frequency is corrected in real‐time for stepwise droplet driving. Taking the *x*‐direction droplet drive as an example, within each control period, Δt, the *y*‐position of the stationary droplet is determined by sweeping frequency for the first 260 ms to identify the center frequency. The droplet is then driven along the *x*‐direction using this frequency for the remaining time, followed by the next cycle of detection‐calibration‐drive (Figure 5c). With control periods of Δt set to 300 and 350 ms, even slight drifts in droplet movement are corrected, ensuring stable progression (Movie S1, Supporting Information). Additionally, the average velocity of the droplet can be changed by adjusting Δt. Shorter Δt reduces the velocity but helps minimize lateral drift (with Δt = 300 ms, the droplet maintains its position within a ±0.25 mm range of its initial *y*‐direction location). This method has been shown to achieve long‐distance, stable transport of droplets with a high success rate (see Note S10, Supporting Information for details). Furthermore, sequential selective driving of multiple droplets has been achieved using the DSAT. Here, the system automatically identified the positions of multiple droplets and selectively drove any one of them based on requirements (Figure 5d). Additionally, when controlling multiple small droplets that are positioned too close to each other, a slight misalignment of the SAW excitation from the center of the target droplet may lead to extreme cases of control failure. This can result in accidental actuation of non‐target droplets or cause the target droplet to shift and merge with a non‐target droplet. A demonstration of these cases can be found in Movie S2 (Supporting Information).

The DSAT is configured with two pairs of orthogonally aligned SFITs, enabling droplet driving and position sensing in both *x* and *y* directions, thereby achieving automated closed‐loop transport of droplets on a plane. Here, experiments on the automatic transport of droplets from their starting to target positions were carried out (Figure 5e). During the first 450 ms of the control period, Δt, the position of the droplet in *x* and *y* directions was detected via frequency sweeping. Based on the detected position and the preset target position, the frequencies and amplitudes of the excitation signals were determined. The remaining time was then used to drive the droplet using these parameters. This cycle of position detection, parameter determination, and driving was repeated in each subsequent period. As shown in Figure 5e, two control strategies for droplet transport were employed and demonstrated, including the segmented path and the direct path strategies (Movie S3, Supporting Information). In the segmented path strategy, the frequency‐synchronized stepping drive method was used to move the droplet along orthogonal paths in two stages. In the first stage, SFITs 1^#^2^#^ were excited to move the droplet along the *y*‐axis to the target *y*‐position. During this process, the *x*‐position was detected to calibrate the excitation frequency, and the *y*‐position was detected to provide feedback on reaching the target. In the second stage, SFITs 3^#^4^#^ were excited to move the droplet along the *x*‐axis to the target *x*, *y*‐position. Here, the detections of the *x*‐and *y*‐positions were used to provide feedback on reaching the target and to calibrate the driving frequency, respectively. The system was programmed to pause driving and detect the position when the droplet was within ±0.3 mm of the target. If two consecutive detection results fell within this range, the system determined that the droplet had reached the target position for that stage or the final destination, and then proceeded to the next stage or stopped. In the direct path strategy, a proportional control method was employed. Based on the detected current position of the droplet, the frequency of the excitation signal was calibrated, and the amplitude of the excitation signal was dynamically adjusted according to the distance to the target (*V*_pp_ = 14–15.5 V, with larger distances require higher amplitudes). Then the SFITs in the *x* and *y* directions were simultaneously activated to move the droplet along a straight line to the target point. These two strategies are suited to different applications. The segmented path strategy enhances operational reliability by activating each SFIT separately, making it particularly effective for single droplet transport due to potential interference among multiple droplets caused by acoustic waves. The direct path strategy requires precise control of signal amplitude to ensure accurate movement direction, but it can effectively avoid interference in multi‐droplet operations, making it suitable for tasks involving droplet merging and other multi‐droplet applications. Finally, automated droplet transport along the trajectories shaped like the letters “H”, “I”, “T” was successfully achieved under closed‐loop control based on position feedback. The frequency‐synchronized stepping drive method, combined with position feedback from acoustofluidic‐based detection, was employed to control the movement of the droplet along segmented paths, progressively driving the droplet to each predefined stage target (Figure 5f; Movie S4, Supporting Information). Image‐based analysis through video tracking confirmed that the center position of the droplet consistently remained within a ±0.35 mm range of the predefined trajectory, as indicated by the dashed lines in Figure 5f.

### 4.3 Feedback‐Enhanced Multifunctional Automated Droplet Manipulation

SAW‐based acoustofluidics can perform various droplet manipulation functions depending on the amplitude and location of the wave‐droplet coupling, including droplet merging, internal mixing, splitting, ejection, and internal particle enrichment, among others.^[^ ^30^, ^36^, ^59^, ^70^ ^]^ The DSAT developed in this study successfully integrates these functions on an acoustic tweezer platform by controlling the position and amplitude of the SAWs generated on a 2D SFITs device, offering a comprehensive acoustic solution for droplet manipulation. Additionally, the closed‐loop control system leveraging the DSAT's droplet sensing capabilities not only supports the automated execution of these functions but also enhances the efficiency and fault tolerance of the manipulation process through real‐time feedback on the positions and numbers of the droplets.

**Figure 6:**
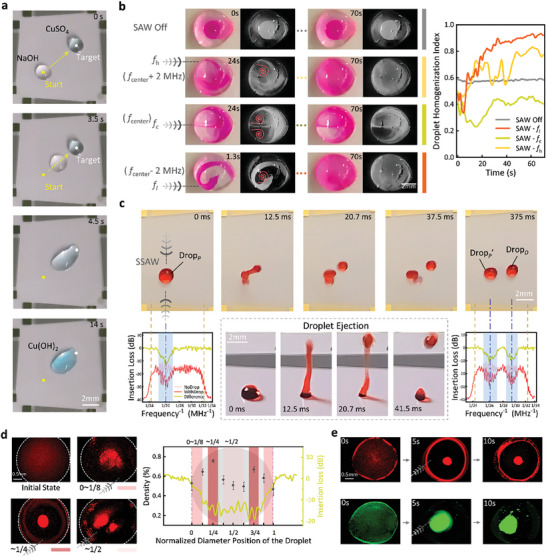
Multifunctional automated droplet manipulation using DSAT. a) Automated merging of CuSO_4_ and NaOH droplets, each with a volume of 2 µL, and their subsequent chemical reaction. *V*_pp_ = 15.5–17 V. b) Mixing of a 2 µL droplet of rhodamine solution (red) in a glycerol droplet (colorless) with a volume of 10 µL. *V*_pp_ = 13 V. Here, the variation curves of the droplet homogenization index under different acoustic field conditions were shown. c) Snapshots of a droplet with a volume of 2 µL (Drop*_P_*), actuated by the TSAWs (*V*_pp_ = 20 V) to eject and split into two 1 µL droplets (Drop*_P’_* and Drop*_D_*). d) Relationship between the particle enrichment effect and the position of the excited TSAWs. The diagram quantitatively analyzed the enrichment of particles with diameter of 5 µm within the 3 µL droplet, where the horizontal axis represented the relative position of the TSAWs acting on the droplet (0, 1/4, 1/2, 3/4, 1), and the vertical axis displayed the particle density of the droplet center area after enrichment. Each position was independently tested three times and each acoustic exposure lasted 10 s. The particle enrichment effect was monitored using an inverted fluorescence microscope. *V*_pp_ = 13 V. e) Automated and optimized enrichment of particles with diameter of 5 µm (red fluorescence) and 20 µm (green fluorescence) within a 2 µL droplet.

Droplet merging is a crucial step for many microfluidic applications based on droplet manipulation, often serving to trigger and initiate chemical reactions.^[^ ^12^ ^]^ Using the DSAT, the automated merging of droplets can be achieved through selective actuation. The automated on‐chip merging of CuSO_4_ and NaOH droplets and the subsequent chemical reaction were demonstrated (**Figure** **6**a; Movie S5, Supporting Information). The DSAT system automatically identified the positions of the two droplets and designated one as the target point. Subsequently, the system employed the direct path control strategy to drive the other droplet to the target droplet, achieving the merge. Once the droplets successfully merged, the driving was halted when the detection system could only recognize one droplet, and a chemical reaction occurred, forming blue Cu(OH)₂ sediment in the merged droplet.

In the field of microfluidic technology, rapid and uniform mixing of viscous fluids is crucial for applications in chemical synthesis, drug development, and diagnostic testing.^[^ ^71^ ^]^ The DSAT integrates the droplet mixing function and leverages the advantages of acoustofluidics to achieve rapid and uniform mixing of viscous fluids. The experiment demonstrated the mixing of a 2 µL droplet of rhodamine solution in a 10 µL droplet of glycerol (Figure 6b). Without the influence of an acoustic field, the rhodamine solution clustered and did not disperse within the glycerol droplet. When TSAWs generated by the SFITs coupled into the glycerol droplet, the induced acoustic streaming enabled uniform mixing of the rhodamine solution within the glycerol. The amplitude of the excitation signals was set to *V*_pp_ = 13 V to ensure only acoustic streaming occurred without moving the droplet. In addition, the droplet homogenization index was used to assess the uniformity of droplet mixing and quantitatively characterize the mixing effect (details in Note S11, Supporting Information). Due to the effective aperture of the TSAWs (≈1 mm) being smaller than the contact diameter of the droplet (≈5 mm), variations in the position of TSAWs excitation relative to the droplet led to different internal flow patterns: i) At the center frequency (*f_c_*), symmetrical acoustic streams were generated, resulting in poor mixing. ii) At the high frequency (*f_h_*), clockwise vortex streaming was generated, aiding in the mixing process despite being weaker. iii) At the low frequency (*f_l_*), anti‐clockwise vortex streaming was induced, which can raise the droplet homogenization index to 0.92 within 70 s, achieving effective uniform mixing (Movie S6, Supporting Information). The experimental results highlighted the critical impact of the excitation position of the TSAWs relative to the droplet on the efficiency of mixing. The DSAT enhanced droplet mixing by precisely controlling the positioning of the excited TSAWs. It achieved this through automatic detection of the position of the droplet and subsequent adjustment of the excitation frequency, optimizing the mixing process.

Droplet splitting is a critical functionality for constructing fluid handling platforms,^[^ ^44^, ^55^ ^]^ and it is integrated into the DSAT developed in this study. As demonstrated in Figure 6c, standing surface acoustic waves (SSAWs) with high amplitude (*V*_pp_ = 20 V) were generated and employed to eject and split a 2 µL droplet into two 1 µL droplets. The process began with detecting the initial position of the parent droplet, followed by the excitation of opposing SFITs at the center frequency to generate SSAWs. These waves were held for 500 ms to induce the ejection and splitting of the droplet, with the resultant droplets falling back onto the substrate. Furthermore, experiments indicated that the ejection‐based splitting process may fail, with the droplet undergoing vertical oscillation without splitting. The DSAT system detected the droplet number after acoustic excitation; if two droplets were detected, the splitting was considered successful, and the excitation would be stopped. If not, the ejection actuation step would be repeated until successful (Movie S7, Supporting Information). This process demonstrated the capability of the DSAT to automate droplet splitting and enhance operational fault tolerance through feedback based on the detection of droplet number.

Particle enrichment within droplets is a key function of droplet manipulation for applications in biosensing and diagnostic testing, offering potential for lab‐on‐a‐chip technologies in point‐of‐care settings.^[^ ^72^ ^]^ Here, the DSAT effectively incorporated this function by generating TSAWs with an RF signal amplitude of *V*_pp_ = 13 V, which induce internal acoustic vortex streaming within the droplet. This streaming caused particles that were slightly denser than the liquid to concentrate at the vortex center due to centrifugal forces, achieving effective enrichment (Figure 6d; Movie S8, Supporting Information). We defined the normalized diameter position of the droplet from 0 to 1, extending from the low‐frequency edge to the high‐frequency edge. The DSAT system detected the edge positions of a droplet containing particles and excited TSAWs at frequencies corresponding to different normalized diameter positions. Experimental results showed that when the TSAWs were applied near the normalized diameter positions of 1/4 and 3/4, particle enrichment was significant. Especially near the 1/4 position, the enrichment effect was optimal due to the larger amplitude on the low‐frequency side. This experiment demonstrated that the relative positioning of the TSAWs to the droplet significantly influenced the efficiency of particle enrichment within the droplet. The DSAT can automatically detect the edge positions of the droplet and adjust the excitation frequency to generate TSAWs at the 1/4 diameter position, achieving optimized particle enrichment. Additionally, the versatility of the DSAT has been demonstrated in handling droplets of different volumes, enabling precise control over particle enrichment in droplets as small as 2 µL (Figure 6e). There is potential to manipulate even smaller droplets by designing SFITs devices with smaller effective apertures.

## 5 Discussion

In this work, we introduced a droplet‐sensing acoustofluidic tweezer (DSAT) platform for multifunctional closed‐loop droplet manipulation. Using the broad frequency response characteristics of the SFITs, DSAT enables the integration of switchable acoustic‐based droplet actuation and acoustic‐based droplet sensing technologies on a single SFITs device. With this integration, multifunctional automated droplet manipulation can be achieved, including droplet transport, merging, mixing, splitting, and internal particle enrichment. We have evaluated the droplet sensing performance of the DSAT, focusing on droplet number, center position, and edge position detection. Using image‐based detection as a reference, DSAT demonstrated high precision and good repeatability in detecting droplet number and positions. The detection of droplet center positions facilitated high‐precision automated droplet transport, while the detection of droplet number and edge positions enhanced the fault tolerance and efficiency of multifunctional droplet manipulation. Experimental results proved that the sensing performance can meet the requirements for multifunctional automated droplet manipulation using acoustic tweezers.

Using the detected droplet positions as feedback, here, we introduced a frequency‐synchronized stepping drive method for droplets with real‐time frequency adjustment, significantly optimizing the performance and automation capabilities of droplet driving using TSAWs. Furthermore, automated closed‐loop transport of droplets along complex planar trajectories was achieved, demonstrating significantly enhanced transport precision compared to the open‐loop droplet transport method using acoustofluidic tweezers previously developed by our group.^[^ ^40^ ^]^ Additionally, the DSAT has integrated multiple droplet manipulation functions, including transportation, merging, mixing, splitting, and internal particle enrichment, fully supporting essential sample handling requirements within the fields of fluid processing and biosensing. By utilizing DSAT's capabilities of sensing droplet number and positions as feedback for closed‐loop control, automated droplet manipulation has been achieved, significantly enhancing the efficiency, precision, and fault tolerance of droplet manipulation.

In summary, the development of DSAT equipped acoustofluidic tweezers with sensing capabilities, responding to the challenges of precision, automation, and functional integration present in existing acoustic droplet manipulation technologies. The compact integration and portability of the DSAT system render it suitable for point‐of‐care testing applications, demonstrating potential for immediate field deployment.^[^ ^73^ ^]^ The integrated capabilities of the platform for multifunctional closed‐loop droplet manipulation provide a novel acoustic solution for fluid handling and biosensing applications.

For future development, we plan to enhance control strategies and elevate the intelligence and automation capabilities of DSAT. We will explore the application of advanced signal processing techniques, such as machine learning, to improve the accuracy of droplet position detection and adaptability to complex droplet scenarios by optimizing algorithms that learn the wave propagation patterns under different droplet configurations. This will enhance the platform's ability to execute multi‐step and complex microfluidic tasks. Additionally, we will explore techniques for multi‐position acoustic excitation on a single platform, forming multiple sets of acoustic tweezers to achieve parallel processing of multiple droplets, thereby expanding the application scope.

## 6 Experimental Section

### Fabrication of the SFITs device

The SFITs were deposited in parallel on a 128° Y‐cut lithium niobate wafer (500 µm thick) by e‐beam evaporation of Cr (20 nm, adhesive layer) and Au (100 nm). Each SFIT features 21 pairs of interdigital electrodes with finger periods ranging from 160 to 120 µm. The resonant frequency bandwidths, determined through testing, for pairs of SFITs are 24.2 to 32.3 MHz for SFIT 1^#^2^#^ and 22.6 to 30.7 MHz for SFIT 3^#^4^#^. Prior to the experiment being carried out, the surface of the substrate has been coated with a layer of fluorosilicone polymer (NEC103, Schanda Co. Ltd., China). After cleaning the substrate, a spin coating approach was used with 10 µL reagent, operating at a speed of 500 rev min^−1^ and then placing the substrate at a standstill at room temperature for 5 min, and then the spin coating was repeated three times.

### Materials

The casings of the droplet actuation modules and droplet detection modules were fabricated using photosensitive resin through 3D printing, and the PCB boards were processed through photolithography, etching, and multi‐layer lamination techniques (by Shenzhen JLC Technology Group Co. Ltd, China). The following chemical reagents were purchased from CODOW: CuSO₄ (2%), NaOH (1%), and glycerol (AR, 99%). Rhodamine B (1%) was purchased from BKMAMLAB. All other droplets consisted of DI water dyed with red dye. Polystyrene particles of 5 µm diameter (fluorescent red) and 20 µm diameter (fluorescent green) were purchased from Jiangsu Zhichuan Technology Co. Ltd, China.

### Instruments

The resonant frequency bandwidths and reference values of the frequency responses of the SFITs devices were measured using a network analyzer (NanoVNA‐H4; NanoLab, China). The droplet motions were recorded by a camera at a speed of 60 fps. Additionally, in the particle enrichment experiments, to facilitate monitoring under an inverted microscope, the SFITs device was mounted on a PCB board using conductive adhesive instead of the contact points. Meanwhile, the droplet detection module was mechanically separated from the droplet actuation module, while maintaining electrical connections. The SFITs device was then placed under an inverted fluorescence microscope (AxioObserver D1; ZEISS, Germany) for particle observation.

### Image‐Based Visual Recognition

Droplet position identification was performed using MATLAB programming, based on grayscale image processing (“rgb2gray”), morphological operations (“imopen”), and connected component analysis (“regionprops”). The calculation of the droplet homogenization index for droplet mixing was conducted using MATLAB, utilizing grayscale image processing (“rgb2gray”), frame differencing, histogram analysis, and entropy calculations. The density calculation after particle enrichment was also conducted using MATLAB, based on pixel recognition and statistical analysis.

## Conflict of Interest

The authors declare no conflict of interest.

## Author Contributions

H.D., Y.A. and J.Z. conceived the idea and designed the research methodology. M.S. and G.M. designed and fabricated the device. M.S. and G.M. performed the experiment and analyzed the data with the help of Z.Y. under the supervision of H.D. and Y.A. M.S. wrote the manuscript, and H.D. and Y.A. contributed to the revision.

## Supporting information



Supporting Information

Supplemental Movie 1

Supplemental Movie 2

Supplemental Movie 3

Supplemental Movie 4

Supplemental Movie 5

Supplemental Movie 6

Supplemental Movie 7

Supplemental Movie 8

## Data Availability

The data that support the findings of this study are available from the corresponding author upon reasonable request.
