# Supplementary material for: Acoustofluidic Tweezers Integrated with Droplet Sensing Enable Multifunctional Closed‐Loop Droplet Manipulation
Source: Adv Sci (Weinh). 2024 Nov 11;12(1):2409394. doi: 10.1002/advs.202409394 (PMC11714172; doi:10.1002/advs.202409394)
Supplement: Supplementary file 1 — Supporting Information [file ADVS-12-2409394-s006.pdf]

ADVANCED SCIENCE
Open Access

Supporting Information

for *Adv. Sci.*, DOI 10.1002/advs.202409394

Acoustofluidic Tweezers Integrated with Droplet Sensing Enable Multifunctional Closed-Loop Droplet Manipulation

Mingyang Sui, Huijuan Dong, Guanyu Mu, Zhen Yang, Ye Ai* and Jie Zhao*

Supporting Information

Acoustofluidic Tweezers Integrated with Droplet Sensing Enable Multifunctional Closed-loop Droplet Manipulation

Mingyang Sui, Huijuan Dong, Guanyu Mu, Zhen Yang, Ye Ai*, Jie Zhao*

M. Sui, H. Dong, G. Mu, Jie Zhao
State Key Laboratory of Robotics and System, Harbin Institute of Technology, Harbin 150001, China
E-mail: dhj@hit.edu.cn

Z. Yang
Institute of Orthopedics, Chinese PLA General Hospital, Beijing Key Laboratory of Regenerative Medicine in Orthopedics, Key Laboratory of Musculoskeletal Trauma & War Injuries PLA, Beijing, 100853, China

Y. Ai
Pillar of Engineering Product Development, Singapore University of Technology and Design, Singapore 487372, Singapore
E-mail: aiye@sutd.edu.sg

This file includes:
Note S1 to S11
Figures S1 to S11
Movies S1 to S8

Note S1. Simulating the frequency response characteristics of SFITs using the COM model

In this paper, we employed the COM model to simulate and analyze the frequency response characteristics of the SAW device.

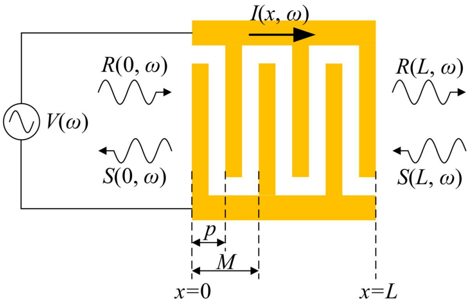

Figure S1. Schematic of the structure of the interdigital transducer (IDT).

First, the uniformly spaced IDT was considered, as shown in Figure S1, characterized by an interdigit spacing of p and a period of M, where M equals $2p$. An excitation voltage, $V(\omega)$, was applied to the IDT, resulting in the amplitudes of the surface acoustic wave (SAW), $R(x, \omega)$ and $S(x, \omega)$, propagating along the x-direction. A current, $I(x, \omega)$, was generated in the IDT. From this configuration, the governing equations of the COM model relating $R(x, \omega)$, $S(x, \omega)$, and $I(x, \omega)$ can be obtained and was shown below [1,2]:

$$\begin{cases} \dfrac{dR(x)}{dx} = -jk_E R(x) + jK_R S(x) e^{-j2k_0 x} + j\alpha_R V e^{-jk_0 x} \\ \dfrac{dS(x)}{dx} = +jk_E S(x) - jK_S R(x) e^{+j2k_0 x} - j\alpha_S V e^{+jk_0 x} \\ \dfrac{dI(x)}{dx} = +j2\alpha_S R(x) e^{+jk_0 s} + j2\alpha_R S(x) e^{-jk_0 x} - j\left(\dfrac{3\omega C_F / M}{3 + j\omega R_F C_F}\right) V \end{cases} \tag{S1}$$

Here, $k_0=2\pi/M$, represents the acoustic synchronization wave number of the IDT. The other parameters are as follows:

$$\begin{cases} k_E = \dfrac{\omega}{v_e} - \dfrac{2\alpha^2 \omega C_F R_F^2 M}{9 + (\omega C_F R_F)^2} - j\left[\gamma + \dfrac{6\alpha^2 R_F M}{9 + (\omega C_F R_F)^2}\right] \\ \alpha_R = \dfrac{3\alpha e^{j\varphi_t}}{3 + j\omega C_F R_F}, \alpha_S = \dfrac{3\alpha e^{-j\varphi_t}}{3 + j\omega C_F R_F} \\ K_R = K e^{j\varphi_g} \cdot \dfrac{j2\alpha^2 R_F M e^{-j2\varphi_t}}{3 + j\omega C_F R_F}, K_S = K e^{-j\varphi_g} \cdot \dfrac{j2\alpha^2 R_F M e^{j2\varphi_t}}{3 + j\omega C_F R_F} \end{cases}$$

Here, v_e represents the equivalent wave velocity of the SAW. α is the transduction coefficient of the IDT, indicating its electro-acoustic conversion efficiency. K is the reflection coefficient. γ represents the propagation loss, which is related to the materials of the electrodes and the piezoelectric substrate. R_F and C_F are the resistance and capacitance per unit period of the IDT,

respectively. For a uniformly spaced IDT, φ_g equals π/2 and φ_t equals π, representing the phase compensations for the reflection grating and electrical potential, respectively.

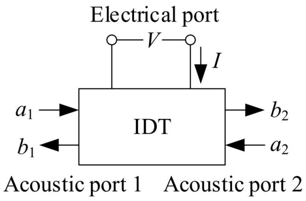

Figure S2. Three-port equivalent circuit model of the IDT.

An IDT can be equivalently represented as a three-port circuit, as shown in Figure S2, which includes two acoustic ports and one electrical port, describing the acoustic and electrical characteristics of the IDT respectively. Assume that the SAW amplitudes entering the two acoustic ports of the IDT are $a_1 = R(x, \omega) = R_I(\omega)$ and $a_2 = S(L, \omega) = S_I(\omega)$, with a voltage, V, applied at the electrical port. The corresponding SAW amplitudes output to the two ports are $b_1 = S(0)$ and $b_2 = R(L)$, and the generated current is I. The solution to the set of COM equations shown in Equation S1 can be written as:

$$\begin{bmatrix} S(0) \\ R(L) \\ I \end{bmatrix} = \begin{bmatrix} P_{11} & P_{12} & P_{13} \\ P_{21} & P_{22} & P_{23} \\ P_{31} & P_{32} & P_{33} \end{bmatrix} \begin{bmatrix} R_I(\omega) \\ S_I(\omega) \\ V \end{bmatrix} \tag{S2}$$

Here, the elements of the P matrix are as follows:

$$P_{11} = \frac{jK_S \sin(DL)}{D\cos(DL) + j\Delta\sin(DL)}$$

$$P_{12} = \frac{D}{D\cos(DL) + j\Delta\sin(DL)} e^{-jk_0 L}$$

$$P_{22} = \frac{jK_R \sin(DL)}{D\cos(DL) + j\Delta\sin(DL)} e^{-j2k_0 L}$$

$$P_{13} = jL\frac{\sin(DL)/2}{DL/2}\left[\frac{\alpha_S D\cos(DL/2) + j(K_S\alpha_R + \Delta\alpha_S)\sin(DL/2)}{D\cos(DL) + j\Delta\sin(DL)}\right]$$

$$P_{23} = jL\frac{\sin(DL/2)}{DL/2}\left[\frac{\alpha_R D\cos(DL/2) + j(K_R\alpha_S + \Delta\alpha_R)\sin(DL/2)}{D\cos(DL) + j\Delta\sin(DL)}\right] e^{-jk_0 L}$$

$$P_{33} = -j2\left(\frac{K_S\alpha_R^2 + K_R\alpha_S^2 + 2\Delta\alpha_S\alpha_R}{D^3}\right)\left[DL - \frac{D\sin(DL) + j\Delta[1-\cos(DL)]}{D\cos(DL) + j\Delta\sin(DL)}\right]$$
$$-2\left(\frac{\Delta(K_S\alpha_R^2 + K_R\alpha_S^2) + 2K_S K_R\alpha_S\alpha_R}{D^3}\right)\left[\frac{1-\cos(DL)}{D\cos(DL) + j\Delta\sin(DL)}\right] + j\frac{3\omega C_F L/M}{3 + j\omega C_F R_F}$$

Here, $\Delta = k_E - k_0$ is referred to as the tuning parameter, and $D = \sqrt{\Delta^2 - K_R K_S}$ represents the dispersion relation of the COM equations. The other parameters, not listed here, are defined by the characteristics of the IDT and conform to the following relationships:

$$P_{21} = P_{12}, P_{31} = -2P_{13}, P_{32} = -2P_{23}$$

Then, all elements of the P matrix can be determined using the equations previously mentioned. In addition, when the ratio of the electrode thickness of the IDT (that is, h_m) to the wavelength of the SAW (that is, λ) is very small ($h_m/\lambda \leq 0.02$), the main parameters of the COM model can be approximately calculated using perturbation theory.

(1) Equivalent wave velocity, v_e

Changes in SAW velocity are primarily caused by electrical and mechanical loading, which are related to the acoustoelectric feedback effect and the mass of the finger electrodes, respectively. Using perturbation theory, the equivalent wave velocity of the SAW, v_e, can be written as:

$$v_e = v_f\left(1 + \frac{\Delta v}{v_f}\right) \approx v_f\left(1 + D_p\frac{K^2}{2} + \eta D_m\frac{h_m}{\lambda}\right)$$

Here, v_f represents the velocity of the SAW. K^2 represents the electromechanical coupling coefficient of the piezoelectric substrate. D_p and D_m are the perturbation coefficients for the wave velocity changes caused by electrical and mechanical loading, respectively, and are given as:

$$D_p = -\frac{1}{2}\left[1 + \frac{P_s(-\cos\eta\pi)}{P_{-s}(-\cos\eta\pi)}\right]$$

$$D_m = \frac{\pi K^2}{\varepsilon_\infty}\left[\left|\frac{u_1}{\Phi}\right|^2(\alpha_1 - \rho_m v_f^2) + \left|\frac{u_2}{\Phi}\right|^2(\alpha_2 - \rho_m v_f^2) - \left|\frac{u_3}{\Phi}\right|^2 \rho_m v_f^2\right]$$

Here, P_s represents the first type of Legendre function, which can be calculated through hypergeometric functions, specifically given by P_s=F($-s$, s+1, 1, 0.5-0.5x), where $s=\omega/v_f\ p/(2\pi)$. Additionally, the dielectric constant ε_∞ is approximately $\sqrt{\varepsilon_{11}\varepsilon_{33} - \varepsilon_{13}^2} \cdot \varepsilon_0$. The mechanical displacement, u_i, and the surface electric potential, Φ, are related to the piezoelectric material used, and ρ_m represents the mass density of the metal electrodes. α_1 and α_2 are the characteristic constants of the metal material. For the 128° YX-cut $LiNbO_3$ and metal electrodes, Au, used in this paper, the respective parameters are K^2=5.5%, $|u_1/\Phi|$ =0.189 nm/V, $|u_2/\Phi|$ =0.014 nm/V, $|u_3/\Phi|$ =0.213 nm/V, ρ_m=19320 kg/m^3, α_1=9.80×10^{10} N/m^2, α_2=2.74×10^{10} N/m^2.

(2) Reflection coefficient, *K*

The interdigital electrodes on the surface of the piezoelectric substrate create discontinuities in the surface impedance, thereby causing reflection of the SAW. The reflection coefficient, *K*, can be approximated as follows:

$$K \approx \frac{1}{p}\left(R_p \frac{K^2}{2} + R_m \frac{h_m}{\lambda} \sin \pi\eta \right)$$

Here, R_p and R_m represents the coefficients for electrical and mechanical loading, respectively. The calculation equations can be given as:

$$R_p = -\frac{\pi}{2}\left[\cos\eta\pi + \frac{P_{1/2}(-\cos\eta\pi)}{P_{-1/2}(-\cos\eta\pi)} \right]$$

$$R_m = -\frac{\pi K^2}{\varepsilon_\infty}\left[\left|\frac{u_1}{\Phi}\right|^2 (\alpha_1 + \rho_m v_f^2) + \left|\frac{u_2}{\Phi}\right|^2 (\alpha_2 + \rho_m v_f^2) + \left|\frac{u_3}{\Phi}\right|^2 \rho_m v_f^2 \right]$$

(3) Transduction coefficient, α

The transduction coefficient, α, represents the electro-acoustic conversion efficiency per unit period of the IDT, can be written as:

$$\alpha = \frac{\bar{\rho}(\beta)}{M}\sqrt{\frac{\omega A K^2}{4\varepsilon_\infty}}$$

Here, $\beta = \omega / v_f$, represents the wave number of the SAW, and $\bar{\rho}(\beta)$ is the average electrostatic charge density of the IDT, can be written as:

$$\bar{\rho}(\beta) = \varepsilon_\infty \frac{2\sin\pi s}{P_{-s}(-\cos\eta\pi)} P_n(\cos\eta\pi), s = \frac{\beta p}{2\pi} - n(0 \le s \le 1)$$

(4) Resistance per unit period of the IDT, R_F

For a uniformly spaced IDT, each interdigital period typically consists of only one pair of finger electrodes, and the electrode resistance per unit period can be written as:

$$R_F = \frac{2 r_s A}{3w}$$

Here, r_s represents the density of the sheet resistance. For a gold film with a thickness of 100nm, r_s is approximately 2.45 mΩ/m^2.

(5) Capacitance per unit period of the IDT, C_F

The capacitance per unit length, C_s, for a pair of interdigital electrodes is:

$$C_s = \varepsilon_\infty \frac{P_{-1/2}(\cos\eta\pi)}{P_{-1/2}(-\cos\eta\pi)}$$

Thus, for interdigital electrodes with an acoustic aperture of *A*, the capacitance per unit period of the IDT, C_F, is:

$$C_F = AC_s$$

For the SAW device composed of a pair of opposed IDTs, as shown in Figure S3 (a), which consists of two uniformly spaced IDTs, IDT$_A$ and IDT$_B$, and a delay distance, L_d. Here, the delay distance can be considered as a three-port circuit with zero electrical parameters at the electrical port and a phase difference at the acoustic ports due to SAW propagation. The three components can then be modeled as three-port circuits, with the acoustic ports connected in series, as shown in the equivalent model in Figure S3 (b).

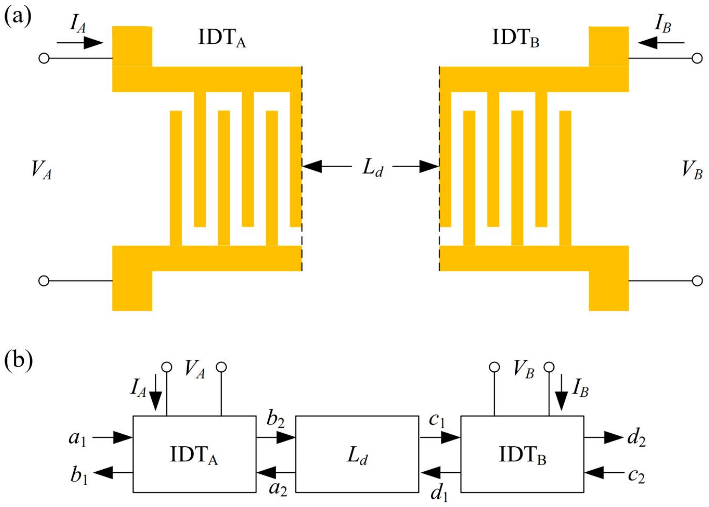

Figure S3. Schematic of the SAW device consists of two uniformly spaced IDTs, and its equivalent circuit model.

By calculating the solutions to the COM equations for each of the three components and combining them, the relationship between the output and input of the entire SAW device can be determined. If reflections at the ends of the surface acoustic wave device are ignored, it can be assumed that a_1=0 and c_2=0. By substituting these boundary conditions into the solutions, the relationship between the current of the SAW device and the input voltage can be obtained and written as [3,4,5]:

$$\begin{bmatrix} I_A \\ I_B \end{bmatrix} = \begin{bmatrix} Y_{11} & Y_{12} \\ Y_{21} & Y_{22} \end{bmatrix} \begin{bmatrix} V_A \\ V_B \end{bmatrix} \quad \text{(S3)}$$

Here, the Y matrix represents the admittance matrix of the SAW device, with its elements as follows:

$$Y_{11} = P_{33}^A + \frac{P_{23}^A P_{32}^A P_{11}^B e^{-j2k_f L_d}}{1 - P_{22}^A P_{11}^B e^{-j2k_f L_d}}$$

$$Y_{12} = \frac{P_{13}^B P_{32}^A e^{-jkL_d}}{1 - P_{22}^A P_{11}^B e^{-j2k_f L_d}}$$

$$Y_{21}=\frac{P_{23}^{A}P_{31}^{B}e^{-jk_fL_d}}{1-P_{22}^{A}P_{11}^{B}e^{-j2k_fL_d}}$$

$$Y_{22}=P_{33}^{B}+\frac{P_{13}^{B}P_{31}^{B}P_{22}^{A}e^{-j2k_fL_d}}{1-P_{22}^{A}P_{11}^{B}e^{-j2k_fL_d}}$$

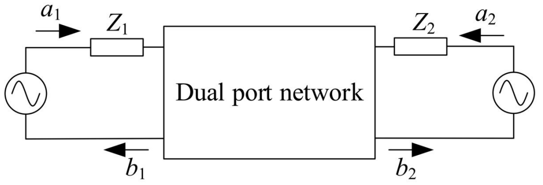

Figure S4. Schematic of the equivalent dual port network for the SAW device.

If the SAW device is modeled as the dual port network shown in Figure S4, where Z_1 and Z_2 are the characteristic impedances of the transmission lines at each end of the device, then the relationship between the reflected powers b_1, b_2, and the incident powers, a_1, a_2, can be defined by the scattering parameters, and can be written as:

$$\begin{bmatrix} b_1 \\ b_2 \end{bmatrix}=\begin{bmatrix} S_{11} & S_{12} \\ S_{21} & S_{22} \end{bmatrix}\begin{bmatrix} a_1 \\ a_2 \end{bmatrix} \quad \text{(S4)}$$

Here, S can be written in terms of the Y matrix as follows:

$$S_{11}=\frac{(1-Z_1^*Y_{11})(1+Z_2Y_{22})+Z_2Z_2^*Y_{12}Y_{21}}{(1+Z_1Y_{11})(1+Z_2Y_{22})-Z_1Z_2Y_{12}Y_{21}}$$

$$S_{12}=\frac{-2\sqrt{R_1R_2}Y_{12}}{(1+Z_1Y_{11})(1+Z_2Y_{22})-Z_1Z_2Y_{12}Y_{21}}$$

$$S_{21}=\frac{-2\sqrt{R_1R_2}Y_{21}}{(1+Z_1Y_{11})(1+Z_2Y_{22})-Z_1Z_2Y_{12}Y_{21}}$$

$$S_{22}=\frac{(1-Z_2^*Y_{22})(1+Z_1Y_{11})+Z_1Z_1^*Y_{12}Y_{21}}{(1+Z_1Y_{11})(1+Z_2Y_{22})-Z_1Z_2Y_{12}Y_{21}}$$

Here, R_i is the real part of Z_i, and Z_i^* is the conjugate complex of Z_i. Here, Z_1=Z_2=50Ω. In the scattering parameters, S_{11} and S_{22} are the reflection coefficients, while S_{12} and S_{21} are the transmission coefficients. Typically, -20log $|S_{11}|$ and -20log $|S_{22}|$ are referred to as the return loss, and -20log $|S_{12}|$ and -20log $|S_{21}|$ as the insertion loss. The variation in insertion loss with frequency represents the frequency response curve of the SAW device.

Unlike uniformly spaced IDTs, Slanted Finger Interdigital Transducers (SFITs) feature varying finger electrode widths, spacings, and periods. The basic structure is illustrated in Figure S5.

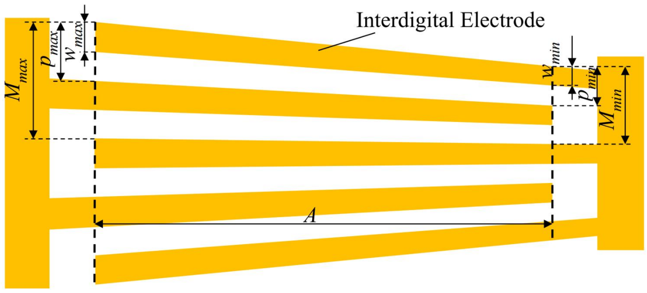

Figure S5. The basic structure of a Slanted Finger Interdigital Transducer (SFIT).

Due to its unique structure, the theories applicable to uniform IDTs cannot be directly used for analyzing the frequency response of the SFIT. To address this issue, the equal aperture separation method is employed to model the SFIT as multiple uniform IDTs. As shown in Figure S6(a), the SAW device consists of two SFITs and a delay distance. The structure of the SAW device, as modeled using the equal aperture separation method, is shown in Figure S6(b). The device is uniformly decomposed into n sub-SAW devices along the direction of SAW propagation, and when n is sufficiently large, the tilt of the SFIT can be considered negligible. Each sub-SAW device consists of two uniformly spaced IDTs, A_i and B_i, and a delay distance. Each sub-SAW device is referred to as a channel of the SFITs device, and by calculating the characteristics of each channel, the properties of the SFIT can be determined.

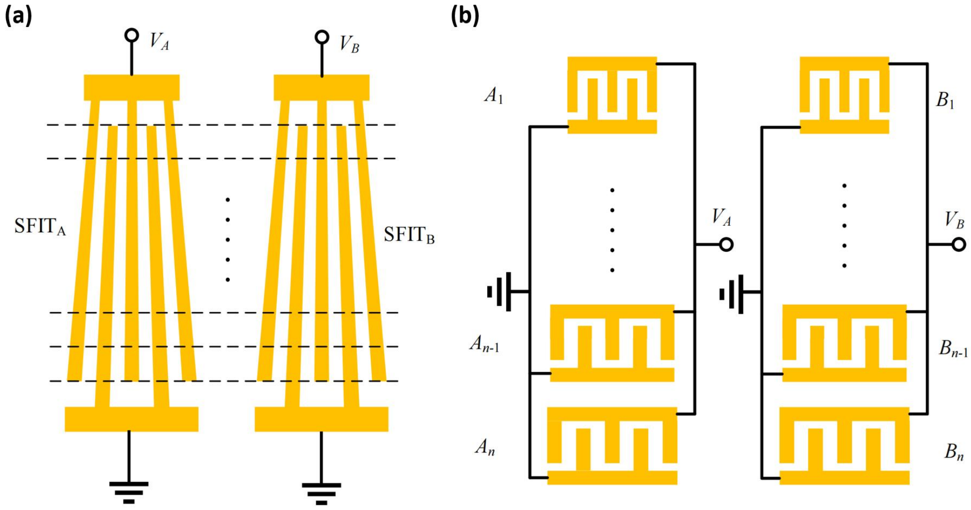

Figure S6. (a) SAW device composed of SFITs. (b) Equivalent structure of the SAW device composed of SFITs.

Each channel of the SAW device satisfies the relationship described by Equation S3, and the channels are connected in parallel. Thus, the relationship between the current of the SAW device shown in Figure S6(a) and the input voltage can be obtained as follows [6]:

$$\begin{bmatrix} \sum_{i=1}^{n} I_A^i \\ \sum_{i=1}^{n} I_B^i \end{bmatrix} = \begin{bmatrix} Y_{11} & Y_{12} \\ Y_{21} & Y_{22} \end{bmatrix} \begin{bmatrix} V_A \\ V_B \end{bmatrix} \tag{S5}$$

Here, the equations for each element of the Y matrix can be written as:

$$Y_{11} = \sum_{i=1}^{n} Y_{11}^i = \sum_{i=1}^{n} \left(P_{33}^{A_i} + \frac{P_{23}^{A_i} P_{32}^{A_i} P_{11}^{B_i} e^{-j2k_f L_d^i}}{1 - P_{22}^{A_i} P_{11}^{B_i} e^{-j2k_f L_d^i}} \right)$$

$$Y_{12} = \sum_{i=1}^{n} Y_{12}^i = \sum_{i=1}^{n} \frac{P_{13}^{B_i} P_{32}^{A_i} e^{-jkL_d^i}}{1 - P_{22}^{A_i} P_{11}^{B_i} e^{-j2k_f L_d^i}}$$

$$Y_{21} = \sum_{i=1}^{n} Y_{21}^i = \sum_{i=1}^{n} \frac{P_{23}^{A_i} P_{31}^{B_i} e^{-jk_f L_d^i}}{1 - P_{22}^{A_i} P_{11}^{B_i} e^{-j2k_f L_d^i}}$$

$$Y_{22} = \sum_{i=1}^{n} Y_{22}^i = \sum_{i=1}^{n} \left(P_{33}^{B_i} + \frac{P_{13}^{B_i} P_{31}^{B_i} P_{22}^{A_i} e^{-j2k_f L_d^i}}{1 - P_{22}^{A_i} P_{11}^{B_i} e^{-j2k_f L_d^i}} \right)$$

After calculating the Y matrix of the SFIT device, its scattering parameters can be determined using Equation S4, thereby obtaining its frequency response characteristics. When droplets are placed on the SAW propagation path of the SFIT device, it can be assumed that the input power to the corresponding channels in the equivalent SAW device structure shown in Supplementary Figure S6(b) is completely absorbed by the droplets. This assumption allows the elements of the Y matrix for these channels to be set to zero. Depending on the size and position of the droplets, the appropriate channels of the Y matrix to be zeroed can be determined. After recalculating the scattering parameters of the device, the frequency response curves of the SFITs with droplets can then be obtained.

Note S2. Numerical simulation of SAW Patterns in SFITs

A simplified three-dimensional model was developed using COMSOL Multiphysics to simulate the surface acoustic wave (SAW) patterns of Slanted Finger Interdigital Transducers (SFITs). This model employs frequency-domain analysis to perform numerical simulations at various frequencies. The simulation results demonstrate that at excitation frequencies of 19.5 MHz, 26 MHz, and 39 MHz, high-amplitude surface acoustic waves are indeed generated at different locations along the transducer. These results validate the capability of SFITs to produce variable wave patterns essential for precise droplet manipulation based on frequency adjustments.

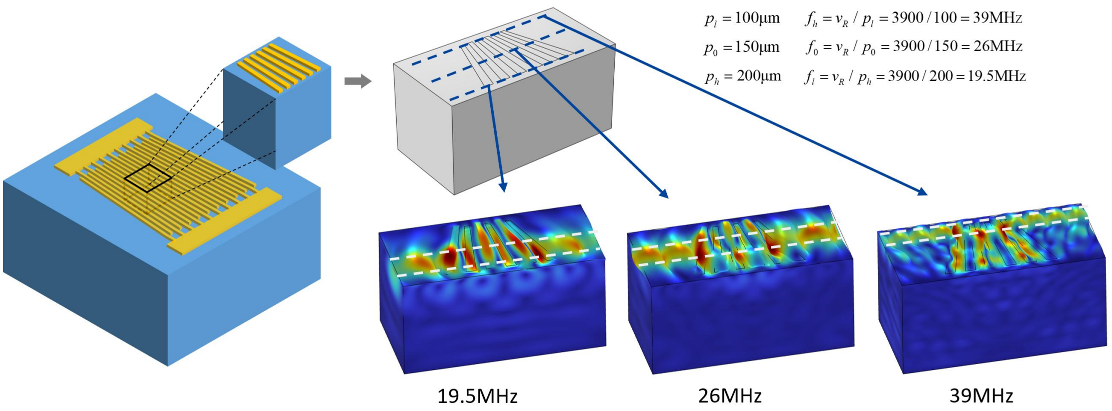

Figure S7. Numerical simulation results depicting the SAW patterns generated by SFITs at various frequencies.

Note S3. Calculation of Effective Acoustic Aperture and Scalability for Larger Droplet Manipulation Areas

Compared to a uniformly spaced IDT, Traveling Surface Acoustic Wave (TSAW) with a narrower beam width can be generated by exciting the SFIT at a certain frequency, and the amplitude can be unevenly distributed along the length of the electrodes. To characterize the width of the TSAW, the effective acoustic aperture of the SFIT, A_{eff}, can be defined and approximated as follows [7]:

$$A_{eff} = \frac{A}{N} \frac{2p_H p_L}{p_H^2 - p_L^2} \tag{S6}$$

Here, N represents the number of finger electrodes pairs of the SFIT, with N=21 for the device designed in this study. A represents the acoustic aperture, set at 6 mm. The maximum and minimum finger periods, p_H and p_L, are 160μm and 120 μm, respectively. Based on these design parameters, the effective acoustic aperture, A_{eff}, is calculated to be 0.98 mm, approximately equal to 1 mm.

Based on Equation S6, we can theoretically evaluate the possibility of expanding the droplet manipulation area. To do this, we need to increase the spacing between opposing SFITs and the overall width of the transducer (acoustic aperture, A). Regarding droplet detection, an increase in A leads to a larger A_{eff}. This enlargement could reduce the variation in insertion loss caused by droplets, as the unobstructed parts of the wave compensate for the loss caused by the droplets, thus diminishing the system's droplet detection capability. Therefore, to keep the effective acoustic aperture almost unchanged, it may be necessary to increase the number of finger pairs or to expand the bandwidth of the SFITs. For our current device configuration, where the acoustic aperture is set at 6mm, increasing the number of

finger pairs to 50 appears feasible, which would enable the acoustic aperture to be extended to 16mm without significantly altering other parameters. In terms of droplet actuation, increasing the spacing requires higher amplitude of SAW to maintain sufficient driving force, which could cause droplets to eject rather than move. This issue can be mitigated by adjusting the excitation voltage across different areas of the device. Overall, with careful design and parameter adjustments, our device theoretically has the potential to manipulate droplets over larger areas.

Note S4. Circuit configuration and working principle of the droplet detection module

The key component of the droplet detection module is an insertion loss detector circuit board, equipped with four radio frequency (RF) interfaces, as shown on the right side of Figure 2d and Figure S8. It is connected via RF cables to the signal output of the droplet actuation module. This module receives and transmits the four RF power signals output by the droplet actuation module to the SFITs through internal circuits and spring contacts. Each signal line is additionally connected to the gain detector chips via an external analog switch. Each pair of SFITs is connected to one gain detector chip, which is used to detect the frequency response of the SFITs resonators and, consequently, the droplets. Here the microcontroller controls the opening and closing of the analog switches, enabling two operational modes: in actuation mode, the switches are open, isolating the gain detector chips and allowing RF signals to pass directly to the SFITs, generating SAWs that actuate the droplets. In detection mode, the switches are closed, integrating the gain detector chips into the signal line; as RF signals are transmitted to the SFITs to generate SAWs for droplet detection, they are simultaneously analyzed by the gain detector chips to obtain the frequency response of the SFITs. Moreover, this droplet detection module is integrated with the SFITs device within the same instrument, requiring only four RF interfaces to receive SAW excitation signals. This integration not only allows it to interface with the droplet actuation module developed in this work but also ensures compatibility with any other SAW generator, offering notable integration and versatility.

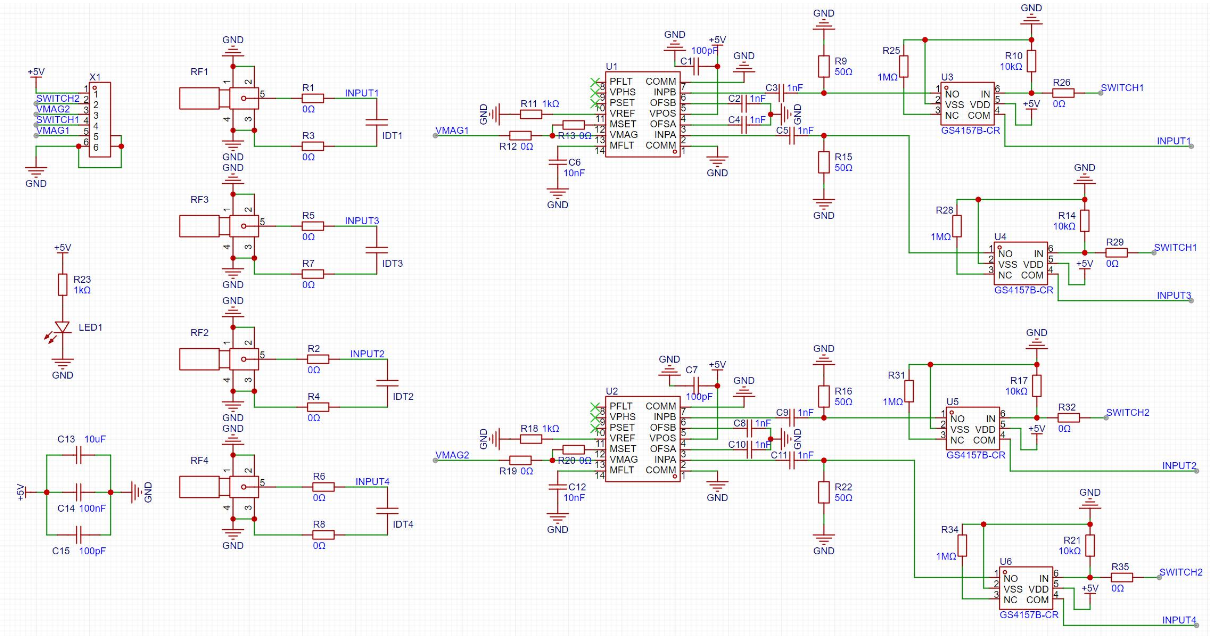

Figure S8. Circuit diagram of the droplet detection module

Note S5. Center-expansion algorithm for determining the number and positions of droplets

In this paper, we introduced a center-expansion algorithm designed for the automatic analysis of frequency response difference data to accurately determine the number and positions of droplets. The search begins at the lower end of the frequency band, firstly identifying the first frequency point where the frequency response difference exceeds -8dB. It then extends from this point toward both lower and higher frequencies until reaching points where the difference equals -3dB, which are considered to mark the edges of the droplet. The center position of the droplet is then determined by calculating the midpoint between these two edge positions. The search continues beyond this droplet on the higher frequency side to locate the next point where the difference exceeding -8dB. This process is repeated to determine the number and positions of all droplets.

It is noteworthy that, according to the test data, even the smallest detectable droplet volume of 0.25 μL exhibits a maximum attenuation point exceeding -8 dB on the frequency response curve. This indicates that within the frequency range corresponding to the position of the droplet, there is at least one difference point where the absolute value is greater than 8 dB. Consequently, -8 dB was selected as the threshold for droplet detection to preliminarily confirm the presence of droplets.

Additionally, -3 dB is commonly used as the threshold value for determining the edges of droplets. However, at the edges of the frequency bands of the SFITs, due to the narrower

beam width of the acoustic waves, greater attenuation is observed in droplet detection. This results in a theoretically lower threshold value for detecting droplet edges compared to the conventional threshold for non-edge areas. Therefore, in this algorithm, the edge detection threshold near the upper and lower ends of the frequency band (for SFIT 1#2# at 24.2-24.8 MHz and 31.2-32.3 MHz, and for SFIT 3#4# at 22.6-23.2 MHz and 29.6-30.7 MHz) is set at -6dB, while it remains -3 dB within other parts of the band. With this configuration, subsequent experiments on droplet edge position measurements have demonstrated good precision and repeatability for the algorithm, with an average deviation of 0.052 mm, a maximum deviation of 0.130 mm, and a standard deviation of 0.024 mm.

Note S6. Detection results of droplet center position using SFIT 1#2#

To evaluate the performance of the multifunctional droplet acoustofluidic platform (DSAT) in detecting the center positions of droplets, droplets were sequentially placed at eight distinct positions along the *x*-axis on the substrate. For each position, the center position of the droplet was first determined using the imaged-based detection method to serve as a reference value. Each position was then independently tested six times by the DSAT, with the results shown in Figure S9, which detailed the frequency response curves for droplet detection at each position. Here, the light red and red curves represented the frequency responses of the SFIT 1#2# without and with droplets, respectively, and the yellow curves showed the differences in frequency response. The blue dashed lines indicated the algorithmically detected centers of the droplets. Statistical analysis revealed an average deviation in detection of 0.038mm (with a maximum deviation of 0.086mm) and a standard deviation of 0.015mm.

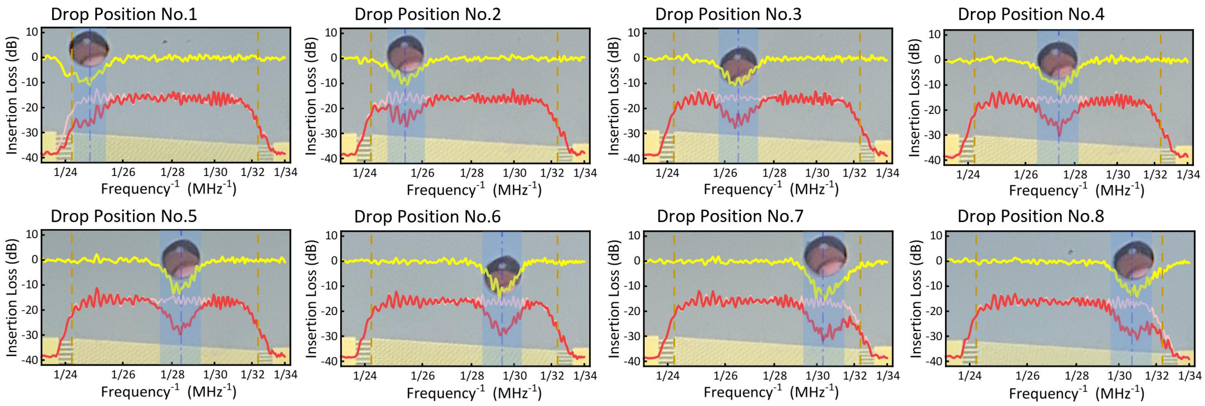

Figure S9. Detection Results for Droplet Center Positions along the *x*-axis.

Note S7. Detection results for edge positions and contact diameters of droplets with various volumes

To evaluate the performance of the DSAT in detecting the edge positions and contact diameters of droplets, droplets of various volumes (0.1, 0.25, 0.5, 0.75, 1, 1.5, and 2 μL) were placed on the substrate. Each volume of droplet underwent six independent tests to measure its edge position and contact diameter, with results displayed in Figure S10(a). Here, the blue double-dashed lines indicated detected droplet edge positions, with the blue areas representing the contact diameters. Overall, statistics showed an average deviation of 0.090 mm for contact diameter measurements (with a maximum deviation of 0.154 mm and a standard deviation of 0.031 mm) and an average deviation of 0.052 mm for edge position measurements (with a maximum deviation of 0.130 mm and a standard deviation of 0.024 mm).

Experimental data for droplets of varying sizes are shown in Figure S10(b). The DSAT can reliably detect droplets larger than 0.25 μL (contact diameter approximately 0.96 mm) with an average deviation of 0.09 mm and a standard deviation of 0.031 mm. For smaller droplets, such as those 0.1 μL in volume, the average deviation increases to 0.164 mm and the standard deviation to 0.147 mm, indicating a marked decrease in both repeatability and accuracy. Additionally, in experiments detecting 0.1 μL droplets, the observed response differences were -8.03, -8.14, -5.75, -9.75, -9.16, and -8.99 dB, with an average of -8.3 dB. This approaches the -8 dB threshold of our automatic detection algorithm and includes instances above this threshold, suggesting a potential for detection failure. Notably, this issue did not occur with droplets larger than 0.25 μL. Therefore, we identify the minimum detectable droplet volume of our developed acoustic tweezers as 0.25 μL, with a contact diameter of approximately 0.96 mm.

a

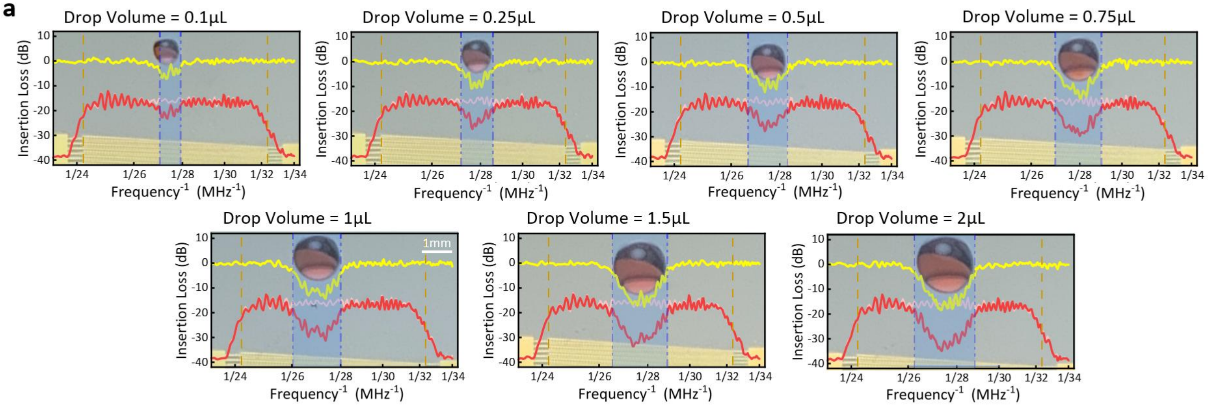

b

Drop Volume	0.1μL	0.25μL	0.5μL	0.75μL	1μL	1.5μL	2μL	
Contact diameter (Image-based) (mm)	0.81	0.96	1.17	1.37	1.52	1.71	1.9	
Contact diameter Test-1 (Acoustic-based) (mm)	0.664	1.050	1.222	1.519	1.579	1.856	2.030	
Contact diameter Test-2 (Acoustic-based) (mm)	0.815	0.975	1.267	1.524	1.664	1.815	1.995	
Contact diameter Test-3 (Acoustic-based) (mm)	0.734	1.046	1.186	1.484	1.579	1.821	2.030	
Contact diameter Test-4 (Acoustic-based) (mm)	0.692	1.081	1.181	1.519	1.585	1.780	2.030	
Contact diameter Test-5 (Acoustic-based) (mm)	0.390	0.975	1.186	1.479	1.616	1.815	1.988	
Contact diameter Test-6 (Acoustic-based) (mm)	0.588	0.975	1.217	1.519	1.616	1.780	2.030	
Average contact diameter (Acoustic-based) (mm)	0.647	1.017	1.210	1.507	1.606	1.811	2.017	Average
Average deviation of contact diameter (Acoustic-based) (mm)	0.164	0.057	0.040	0.137	0.086	0.101	0.117	0.09
Standard deviation of contact diameter (Acoustic-based) (mm)	0.147	0.048	0.033	0.020	0.033	0.029	0.020	0.031

Figure S10. (a) Detection results for edge positions and contact diameters of droplets with various volumes. (b) Experimental data for droplets of varying sizes. Our system can reliably detect droplets larger than 0.25 μL with an average deviation of 0.09 mm and a standard deviation of 0.031 mm. For smaller droplets, such as those 0.1 μL in volume, the average deviation increases to 0.164 mm and the standard deviation to 0.147 mm.

Note S8. Detection results of droplet center position using SFIT $3^{\#}4^{\#}$

To evaluate the performance of SFIT $3^{\#}4^{\#}$ in detecting the center positions of droplets along the *y*-axis, droplets were sequentially placed at five distinct positions along the y-axis on the substrate. Each position was independently tested six times by the DSAT, with the results shown in Figure S11. In the first five diagrams, the light green and green curves represented the frequency responses of the SFIT $3^{\#}4^{\#}$ without and with droplets, respectively, and the yellow curves showed the differences in frequency response. The final diagram displayed the comparison between the results obtained by the acoustic method and the image-based method. The average deviation for detection of the center position in the *y*-axis was 0.066 mm, with a maximum deviation of 0.192 mm, and a standard deviation of 0.020 mm.

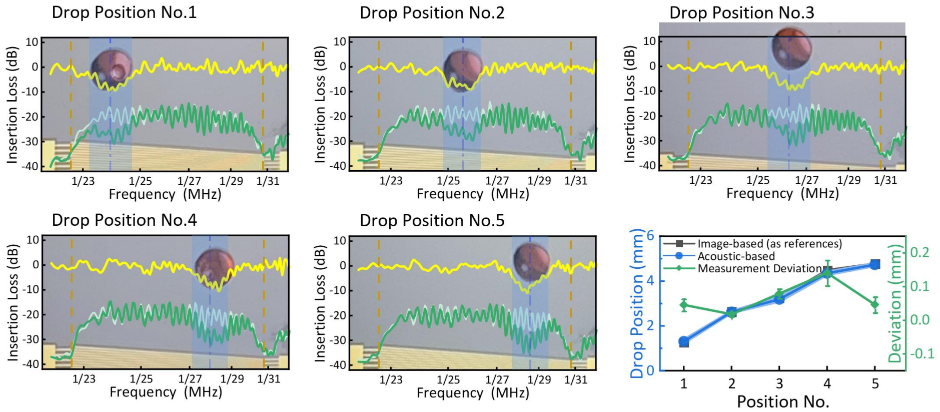

Figure S11. Detection Results for Droplet Center Positions along the *y*-axis.

Note S9. Resolution of droplet position detection using SFITs

The determination of droplet positions by DSAT is based on the analysis of the detected frequency data. Consequently, the resolution of position detection can be influenced by the frequency sweep step size, Δf. According to the function relating frequency to position as described in Equation 1 in the main text, the position detection resolution, ΔP, can be calculated as follows:

$$\Delta P_i = \frac{A \cdot f_{L,i} \cdot f_{H,i}}{f_{H,i} - f_{L,i}} \cdot \frac{1}{f_i^2} \cdot \Delta f$$

Here, ΔP_i represents the resolution of droplet position detection along axis i (x or y), f_i represents the current frequency, and A represents the aperture, which is 6mm in this study. $f_{L,i}$ and $f_{H,i}$ respectively represent the lower and upper frequency limits for the SFITs on axis i. It can be seen that the smaller the frequency sweep step size, the lower the numerical value of the resolution, indicating a higher resolution. Furthermore, since the resolution is frequency-dependent, the detection resolution varies at different positions along the electrodes. For example, when the step size is set to 0.05 MHz, for SFIT $1^{\#}2^{\#}$, the resolution at the lower frequency limit is approximately 0.0493 mm, while at the upper frequency limit, it is about 0.0278 mm, indicating that the resolution varies between 0.0278 and 0.0493 mm. For SFIT $3^{\#}4^{\#}$, the resolution ranges from 0.0273 and 0.0503 mm.

Note S10. Comparison of effects of long-distance stable droplet transport driven by SFITs using different methods

Here, we implemented three different methods to adjust the excitation frequency for driving droplets over long distances using SFITs. The first method involved manually observing and setting the excitation frequency, then continuously activating the SFIT to generate TSAWs to

drive the droplet, as shown in Fig. 5a of the main text. The second method involved automatically detecting the center frequency using the DSAT before driving the droplet, followed by continuous excitation of the SFIT at this frequency, as shown in Fig. 5b of the main text. The third method employed a frequency-synchronized stepping drive method to adjust the excitation frequency applied to the SFIT based on the real-time position detection of the droplet, as shown in Figure 5c of the main text. Each method was carried out in ten distinct experiments, with successful long-distance transport defined as the droplet traveling more than 6mm along the *x*-axis. Experimental results showed success rates of 30%, 60%, and 100% for the first, second, and third methods, respectively, demonstrating the superior efficiency of the frequency-synchronized stepping drive method in ensuring stable long-distance transport of droplets.

Note S11. Calculation of the droplet homogenization index in droplet mixing experiments

In the droplet mixing experiments, we introduced a droplet homogenization index to quantitatively evaluate the uniformity of the mixing. This index was calculated based on the normalized entropy values of the grayscale histograms. Specifically, by conducting a frame-by-frame analysis of the video of the droplet mixing, starting from the initial frame (used as a reference image), one frame per second was extracted to monitor the mixing progress over time. Each frame was first converted into a grayscale image, and a difference image was calculated against the reference image. These difference images can effectively highlight the regions of change caused by mixing. The grayscale histogram of each difference image was calculated and normalized, and the entropy value, *E*, can be calculated and written as:

$$E = -\sum_{i=0}^{L-1} (\ )\log_2 p(\ )$$

Here, $p(i)$ represents the normalized histogram value for the ith gray level. L represents the number of gray levels. For an 8-bit grayscale image, which contains 256 gray levels, L equals 256. A higher entropy value indicates that the image content is more uneven; conversely, a lower entropy value suggests that the image content is more uniform and ordered. To more intuitively represent the uniformity of the mixing, we used the maximum theoretical entropy value, E_{max}=log2(L), as a reference, and defined the droplet homogenization index, U, as follows:

$$U = 1 - \frac{E}{E_{\max}}$$

The original values of the homogenization index ranged from 0.15 to 0.25. For ease of analysis, we applied a linear transformation to normalize these values to a range of 0 to 1. A higher droplet homogenization index indicates better mixing performance, where values approaching 1 signify high uniformity, and those close to 0 indicate poor mixing. This parameter provides an intuitive and effective means to quantify and describe the experimental results.

Supplementary References:

[1] Dong-Pei Chen, H. A. Haus, *IEEE Trans. Son. Ultrason.* **1985**, *32*, 395.

[2] B. P. Abbott, C. S. Hartmann, D. C. Malocha, In *Proceedings., IEEE Ultrasonics Symposium*, IEEE, Montreal, Que., Canada, **1989**, pp. 129–134.

[3] P. S. Cross, R. V. Schmidt, H. A. Haus, In *1976 Ultrasonics Symposium*, IEEE, **1976**, pp. 277–280.

[4] Abbott B P, *PhD Thesizs*. University of Central Florida **1989**.

[5] S. Datta, B. J. Hunsinger, *Journal of Applied Physics* **1980**, *51*, 4817.

[6] T.-T. Wu, I.-H. Chang, *Journal of Applied Physics* **2005**, *98*, 024903.

[7] X. Ding, J. Shi, S.-C. S. Lin, S. Yazdi, B. Kiraly, T. J. Huang, *Lab Chip* **2012**, *12*, 2491.

Description of Supplementary Movies:

File Name: Movie S1

Description: **Droplet movement using frequency-synchronized stepping driving method.** Control periods were set at 300ms and 350ms, respectively. Scale bar: 2mm.

File Name: Movie S2

Description: **Sequential selective driving of multiple droplets.** The initial detection center frequencies were 25.10 MHz, 28.25 MHz, and 31.35 MHz, and the droplets were driven near these respective frequencies. Scale bar: 2mm.

File Name: Movie S3

Description: **Droplet transport on the substrate.** Two control strategies were employed respectively, including the segmented path strategy and the direct path strategy. Scale bar: 2mm.

File Name: Movie S4

Description: **Automated droplet transport along 'H', 'I', 'T' trajectories under position feedback closed-loop control.** The movement of the droplet was controlled along segmented paths, progressively driving it to each predefined stage target. Scale bar: 2mm.

File Name: Movie S5

Description: **The automated on-chip droplet merging process.** The system automatically identified the positions of the two droplets and drove one towards the other. Scale bar: 2mm.

File Name: Movie S6

Description: **The automated droplet mixing process.** The frequencies were set as f_h, f_c, and f_l applied at specific positions relative to the droplet. Scale bar: 2mm.

File Name: Movie S7

Description: **The automated ejection-based splitting of a 2μL droplet.** The waves were held for 500ms to induce the ejection and splitting of the droplet. Scale bar: 2mm.

File Name: Movie S8

Description: **Enrichment of 5μm particles within a 3μL droplet.** After the droplet position was automatically detected, the TSAWs were applied near the normalized diameter positions of 1/4 and were continuously activated for 10 seconds. Scale bar: 500μm.
